# Non quasi-Hemispherical Seismological Pattern of the Earth’s Uppermost Inner Core

**DOI:** 10.1038/s41598-018-20657-x

**Published:** 2018-02-02

**Authors:** Marian Ivan, Rongjiang Wang, Rami Hofstetter

**Affiliations:** 1 University of Bucharest, Bucharest, Romania; 2 Helmholtz Center Potsdam, GFZ German Research Center for Geosciences, Potsdam, Germany; 3 Geophysical Institute of Israel, Lod, Israel

## Abstract

We assembled a database consisting of 5,404 PKIKP/PKiKP observations from 555 events, where PKIKP is the phase sampling the inner core (IC) and PKiKP is the phase reflected at the inner core boundary (ICB). Around 138° distances, their differential arrival times and amplitude ratio are mostly sensitive to the seismic velocity and attenuation structure in the uppermost IC (UIC), respectively. Our observations do not support a large-scale anisotropy in the UIC, but do not exclude its presence in some restricted areas. A robust inversion for the isotropic P-wave velocity perturbations shows a higher velocity cap with a radius of ~60°, approximately centered beneath the Northern Sumatra, with a local low velocity zone beneath the central Indian Ocean. The rest of the UIC, including the Northern part of Eurasia and of the Atlantic Ocean, exhibits mostly lower velocity. Amplitude ratio values of PKIKIP/PKiKP (observed vs. computed) from 548 high signal-to-noise (>5) recordings show a large variance, suggesting only a faint correlation between higher velocity and lower attenuation in the UIC. Our results provide better constraints to the models invoking a heat transfer in the UIC, with a complex temperature pattern near ICB.

## Introduction

The internal structure and composition of the Earth’s inner core (IC) provides realistic conditions for the geodynamo and is essential for the understanding of the outer core (OC) and of the mantle dynamics^1^. Therefore, it is important to accurately map the spatial distribution of P-wave velocity and attenuation, especially in the uppermost IC (UIC, roughly the top 100 km beneath the IC boundary). Seismological evidence suggests a quasi-hemispherical dichotomy of the UIC^2,3^, with a meridian separation. It is considered that the quasi-Eastern hemisphere (qEH, commonly assumed from 40° E to 180° E) displays a higher velocity (and lower attenuation) with respect to a 1-D global reference model. The opposite situation is assumed for the quasi-Western hemisphere (qWH, from 180° W to 40° E). Various other studies obtained slightly different boundaries between the two q-Hs, with possible variations of the above limits with depth^4,5^. Anisotropy in the UIC is also questionable, from an isotropic or weakly anisotropic UIC^3,6–8^, (especially in the qEH), to complex models involving the variation of anisotropy and/or attenuation with depth at the top of the IC^9–11^. Yet, the results related to the UIC anisotropy beneath Africa^9^ are obtained near a presumed boundary of the two q-Hs, where heterogeneity is routinely difficult to separate from anisotropy.

Attempts to link the available seismological observations to thermal effects near the ICB also considered a (quasi)-hemispherical IC^12^. Differential arrival times of phases with similar path in the mantle are routinely assumed to be related to the lateral variations of waves’ velocity in the proximity of ICB and/or to irregularities of ICB topography^13^. Such disturbances with respect to a 1-D global reference model are assumed to be an effect of the lateral variations in temperature or composition of the UIC, but the exact relation chemistry, freezing (or melting) and the velocity/attenuation /topography perturbations cannot be resolved directly by seismological observations^14^. For example, a convection model of the inner core^1,12^ predicts freezing in the qWH, centered at G (growth) point around (0, 80°W), and melting in the qEH (around M point, near (0, 100°E). Alternatively, heat is extracted from the outer core by the vigorous mantle convection, large enough to allow heat flowing into the inner core^15,16^. In such a model, freezing is dominant in the qEH, while a large zone of melting is located in the qWH. At least in some areas, a correlation between the core-mantle velocity pattern and the ICB one is expected. Some thermal models^12^ require (at least) a degree one spherical harmonic pattern, as described by a (linear) variation in cos(Δ) (where Δ is the angular distance of the PKIKP bouncing point to a certain cold or warm pole). While some observations^12^ show a linear variation in Δ only, other data sets^17^ suggested a sharp transition between the two qHs, compatible with models of a translating inner core^18^.

Recently, some local departures from a longitude line separation between the two q-Hs have been reported, challenging the q-H pattern for the deeper part of the core^5,11,19–23^. To accurately identify their spatial position, a proper knowledge of the velocity pattern in the UIC is needed as well.

## Data and preliminary processing

To explain some of the above discrepancies, we analyzed a large data set of high quality PKIKP/PKiKP global observations from 553 earthquakes and two nuclear explosions (1993, October, 5^th^ and 1996, January, 27^th^). We select mostly intermediate to deep depth events to minimize signal disturbances by the depth phases and short-period scattering effects. Some good recordings from shallow earthquakes are also used, but carefully examined to avoid misinterpretation of the PKiKP by PKIKP depth phases. Earthquakes with a complex source-time function are discarded. The entire list of events can be found as Supplementary Table S1. The observations are made in the distance range 133°–142°. At shorter distances, the arrival times of the two phases are too close to each other. At greater distances, the signal is usually severely disturbed by the high amplitude of the short-period precursors (PKPpre, or PKhKP) anticipating the PKIKP arrival, due to the increasing amplitude of PKP_Bdif near B-caustic point around 145°.

**Figure 1:**
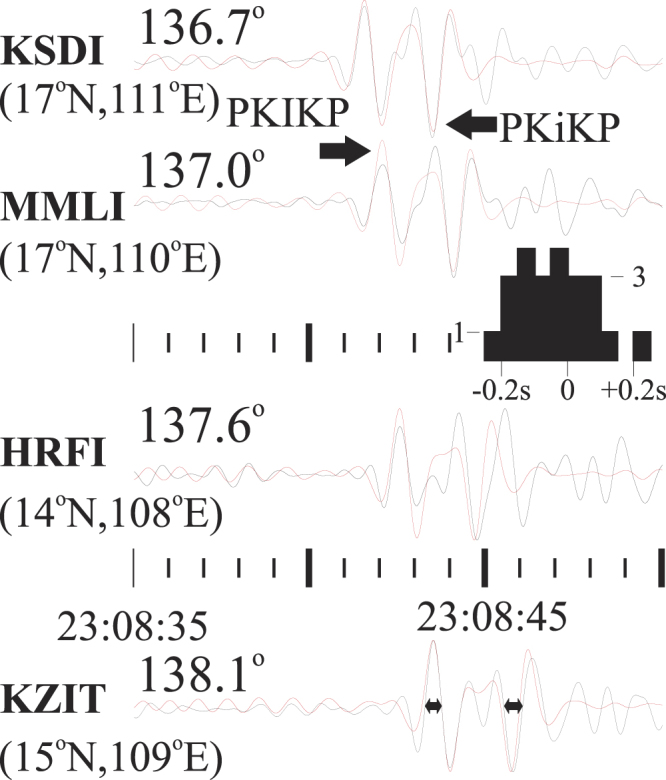
Recordings at some Israel Seismic Network stations of 2013, April, 13^th^ Vanuatu event. A zero-phase Butterworth band-pass filter in the range 0.7 to 2 Hz has been applied both to the broad-band vertical recordings (black) and to the corresponding synthetics (red). Epicentral distances are shown. The location of the PKIKP bouncing points (indicated in the parenthesis) is in the very proximity of the central meridian (110°E longitude) of the quasi-Eastern Hemisphere. Yet, all the differential times are very close to zero. The inset histogram is obtained for the 23 differential times at the stations in Turkey for the same earthquake, and for the 2017, April, 5^th^ Vanuatu event. The bouncing points are in the same area. The mean of the differential times is −0.05 +/− 0.05 seconds (95% confidence error), significantly different from a value of 0.65 seconds obtained from a velocity perturbation around 1% (in respect to ak135 model) as suggested from the q-EH model^49^. The time windows used to obtain the differential times at KZIT station are approximately indicated by the double horizontal arrows.

Synthetic seismograms are used as reference, calculated using the code QSSP^24^ based on the ak135 global Earth model and Global Centroid Moment Tensor source parameters^25,26^. If available, the earthquake locations are taken preferably from the ISC Bulletin^27,28^. Some examples of recorded and synthetic core phases are shown in Fig. 1. The differential times are estimated by cross correlation between the recorded or synthetic phases for time windows of ~0.5 s length, starting immediately following the wave onset on the broad-band, vertical recording. A zero-phase Butterworth band-pass filter in the range 0.7 Hz–2 Hz has been applied^29^, to the recordings and synthetics. We estimate the accuracy in the evaluation of PKiKP vs. PKIKP (O-C, observed minus computed) to be better than 0.1 seconds. This accuracy estimation is supported by the estimations made at a large number of highly confined stations from the same array or local network. The PKIKP phases recorded at these stations are sampling almost the same region of the IC.

**Figure 2:**
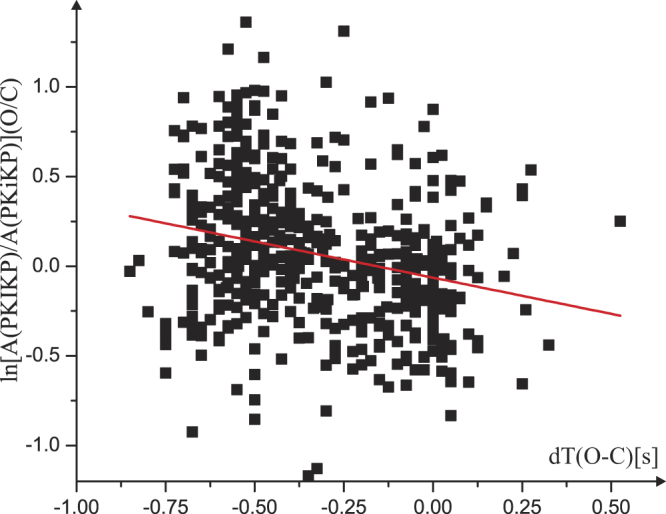
Natural logarithm of the amplitude ratio values of PKIKP/PKiKP (Observed/Computed) vs. the differential times from 548 high (>5) signal-to-noise observations. R-square value of the linear regression is equal to 0.08, indicating a large variance in the data (R-squared = 1 for a linear correlation).

Amplitudes of both PKIKP and PKiKP might be disturbed if short-period precursors are significant in the recordings. We select 548 recordings with the PKIKP at least five times larger than the maximum amplitude of the forerunners observed in a 12 second time window anticipating the PKIKP onset. The amplitude ratios values of PKIKP/PKiKP (Observed/Computed) show a large variance, in many cases observed for stations of the same array or local network (Fig. 2). For example, 19 observations at the Warramunga array of the 2007, September 26^th^ Ecuador event provided an average differential time of −0.44 +/− 0.03 seconds (95% confidence error) and an average natural logarithm (NL) of the amplitude ratio equal to 0.09 +/− 0.08. The PKIKP paths sampled the UIC in the Southern Pacific. In the case of the 2017, June, 24^th^ South Peru event, 22 observations at the same array show −0.54 +/− 0.02 seconds for the differential time, but 0.76 +/− 0.05 for the NL amplitude ratio. Similar variance of the amplitude ratio (or even greater) is routinely observed at stations in Kazakhstan but also at the GRF and YKA arrays. It indicates that IC regions with much closed velocity values may show large variance in attenuation. In particular, 18 observations at the Central Asia stations BRVK, BVAR, CHKZ and VOS from 12 South Sandwich earthquakes show the differential times close to zero (a mean value of 0.02 +/− 0.04 s) and an average NL of the amplitude ratios of 0.05 +/− 0.10. The highly confined ray paths are centered beneath (0°, 30° E).

## Results

**Figure 3:**
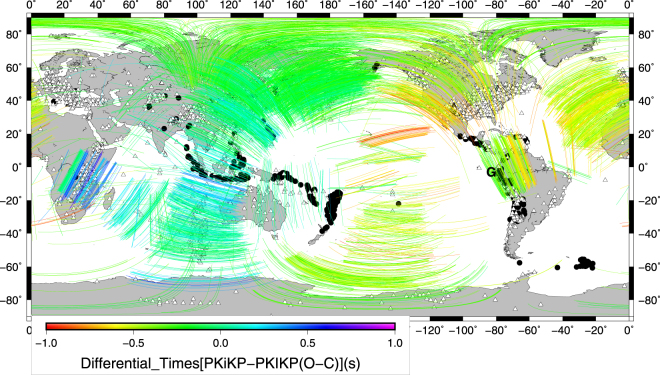
Differential times of PKiKP-PKIKP (O-C) observations. Triangles indicate the seismological stations and black filled circles are the epicenters. Colored lines correspond to the PKIKP paths into the inner core. Pont G at (0°, 80°W) is indicated. Figure produced with Generic Mapping Tools (GMT 5.1.2)^48^.

In general, the differential times (O-C) of PKiKP-PKIKP show some resemblance to the quasi-hemispherical pattern (Fig. 3), but important differences can be also noticed. Recordings of Banda Sea (or Java) events at stations in the NE Canada and United States systematically show differential PKIKP/PKiKP times around −0.2 seconds, despite the corresponding rays sample the qEH beneath the Northern Pacific, Siberia or Central part of Northern Eurasia. The same situation is seen for the earthquakes in South Pacific (e.g. Papua New Guinea) observed at stations in Spain/Portugal, for most of the Vanuatu events observed at stations in Israel or Turkey, or for the Solomon Islands earthquakes observed in Germany and France. Such observations do not support the quasi-hemispherical dichotomy of the IC with a certain longitude line separation, at least along 180° meridian.

There are no areas in Fig. 3 showing a large number of rays at a broad range of orientations to display a pattern reliably suggesting the presence of anisotropy at large scales. Most of Eastern Asia is highly sampled by 245 core phases from 25 Vanuatu events recorded by various stations located in Egypt, Israel, Turkey and Europe. Most of the corresponding differential times are close to zero (around −0.1 to −0.2 seconds). There is no pattern suggesting the presence of anisotropy here, in spite of the broad ray orientations, from a near equatorial one (when sampling IC beneath Indochina) to a near polar path (beneath Kamchatka). The rays sampling the IC beneath Australia and surrounding areas are oriented both on a North-South direction and parallel to the Equator. Yet, all the corresponding differential times are close to zero. They suggest the absence of UIC anisotropy here^30^. Beneath the North Pacific, there are rays with both near equatorial and near polar paths, but all the differential times show slightly negative values. The last observation suggests not only the lack of anisotropy here, but also the absence of a q-H separation at high latitudes around 180° meridian.

A more complex pattern is seen beneath South Africa. In the framework of a q-H IC, such observations have been considered as a result of a complex IC, displaying important vertical and horizontal perturbations in the (isotropic) velocity, anisotropy and attenuation^9,10^. However, rays at different orientations do not sample exactly the same volume of the IC. There are basically two orientations only, a quasi-equatorial and a quasi-polar one. While many quasi-polar observations (of higher quality) are available, there are only a limited number of equatorial ones (of low to moderate quality). It is difficult to accurately estimate here the three parameters describing the anisotropy.

A similar situation is observed beneath the Northern part of the Southern America, where all the observations are near polar ones, showing a regional horizontal gradient in the differential times^31^. Consequently, it is hard to discriminate here between anisotropy and heterogeneity too^32^.

## Inversion Results and Discussion

For a station at 138° distance, both PKIKP and PKiKP at 1 Hz have the Fresnel diameter along ICB around 19°. At the PKIKP ray turning point, the vertical Fresnel diameter exceeds 250 km^33^. The ability of PKIKP to resolve structures at a scale much below the above values, both horizontally and vertically, is questionable. The use of the synthetic seismograms obtained with 1-D model in such complex areas is problematic too, especially when the ray turning point is considered as being representative for the whole ray path of PKIKP in the IC. For a PKIKP recorded at 138 degrees, the last approach may introduce its own errors in the location of the velocity perturbation as large as 16 degrees in the horizontal direction.

Consequently, we prefer to interpret the differential time values as a result of heterogeneity rather than of anisotropy, without excluding that anisotropy could be present in some restricted areas. We divide each of the two uppermost layers of the IC in the ak135 model into cells of 20 ° × 20 ° degrees. The travel time corresponding to the IC leg of PKIKP is around 65 seconds and the observed differential times are in the range −1.0 to 0.7 seconds. So, we invert the differential times assuming that the isotropic velocity perturbations are in the range −1.2 to 1.2%. The inversion results are interpolated by kriging method with low smoothing^34^. Such an approach is more able to preserve the strong lateral gradients^35^, if existing in the UIC structure. However, it is also expected to produce some small, detailed patches not necessary entirely supported by the input data, which are routinely removed by various interpolation methods and/or supplemental filtering^36^. A checkerboard test shows good resolution results (see Figures S1, S2 in the Supplemental Material), especially for the cells hit by more than 43 rays (around 5% of the maximum number of rays crossing a cell, which is 862). The differential time values are explained as a result of velocity perturbations along the whole path of PKIKP in the IC. Inversion is done for the two vertical layers above only to seek consistency with the ak135 model, thoroughly used in this study. So, any significant vertical variations of velocity perturbation obtained between the two layers in our inversion should be regarded with care, given the Fresnel diameter of PKIKP.

**Figure 4:**
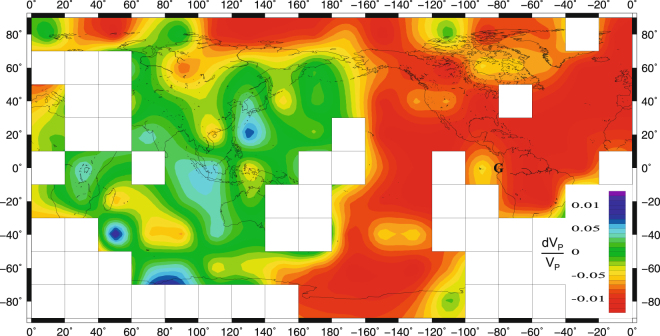
Velocity perturbations at the top of the inner core (first layer beneath ICB in ak135 model, depth from 0 to 51.11 km beneath ICB). Data have been interpolated with kriging method^34^ and a low smoothing. White squares show the areas where the small number of rays did not allow reliable results. The model explains more than 80% in the initial variance (evaluated for null velocity perturbations). Point G at (0°, 80°W) is indicated. Figure produced with Generic Mapping Tools (GMT 5.1.2)^48^.

The inversion results explain more than 80% of the data variance. They show that UIC is mainly represented by a low velocity cap (Fig. 4 and Supplemental Fig. S3) extended beneath most of the so-called qWH, but also beneath the northern part of Eurasia. A local maximum around (0°, 90°W) is mainly the result of a north-northeast to south-southwest gradient in the observed differential times. A similar pattern has been observed in the same area for PKiKP vs. PcP amplitudes (the last phase is the P wave reflected by the core-mantle boundary). This has been explained by a patch of mushy material of a few kilometers high, with a gradual change from the outer core to the IC^37^. This could also explain the extinction (very low amplitudes) of PKiKP bouncing ICB in that area, for some of the South Sandwich events recorded by the Canadian stations (Supplemental Fig. S4).

The rest of the UIC, which is more heterogeneous, is mainly represented by a higher velocity cap with a radius around 60°, roughly centered beneath the northern Sumatra. There are several local maxima here. The first one, placed beneath Sumatra, is the result of south Pacific events observed at the African stations. A second one is located beneath the southeast Africa (0°, 30°E), probably extended toward the southern part of the Indian Ocean. It is mainly the consequence of various observations of South Sandwich events recorded by Central Asian stations. The local maximum around (20°N, 130°E) is suggested by various recordings at European stations of the South Pacific earthquakes. Other local maxima around (0, 90°W) and (40°S, 140°W) are well supported too by the corresponding differential times observations. A low velocity area is observed beneath the central Indian Ocean around (40°S, 80°E), likely to be extended towards NorthWest, in agreement with other previous observations^38^. Irrespective the checkerboard test results, we estimate the rest of the small patches are less supported, being most likely a result of the adopted parametrization and, especially, of the kriging interpolation with low filtering.

There are a few negative differential times in Fig. 3 for rays beneath southwest Africa, observed for some Sandwich events recorded at the Asian stations. The number of rays crossing this area is too small to allow a definite conclusion. A low-velocity volume in the UIC beneath southernmost Africa could be an alternative possible explanation to the anisotropy in that region^9^, suggesting the presence of a convective cell here^16^, could be correlated with an anomalous low-velocity zone near core-mantle boundary, present in various tomographic models^39–41^.

Given the above comments about the Fresnel zone, we also use a single-layer (~100 km thick) model in inversion. The results (Supplemental Figures S5 and S6) are quite similar to the one obtained for the two-layers inversion. All of them suggest the low velocity zone beneath Central Indian Ocean and the local maximum around (0°, 90°W) have a limitted extend in depth beneath the IC boundary.

**Figure 5:**
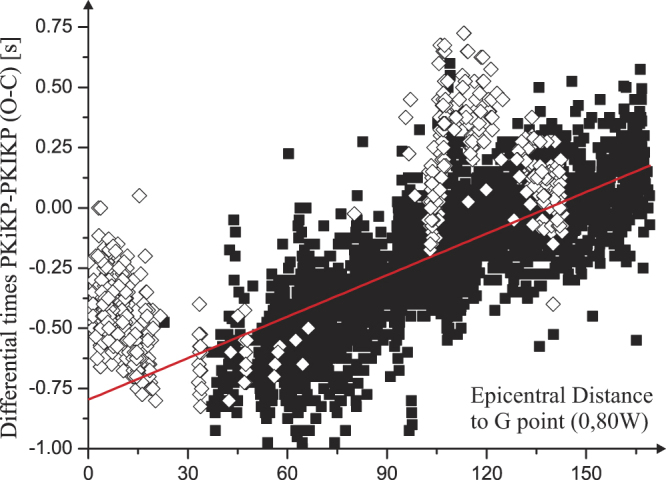
Differential times PKiKP-PKIKP vs. angular distance of the PKIKP bouncing point to the (growth) G point (0, 80°W). Significant departures from the regression line can be observed near 15° or 120° distances due to some of the observations related mainly to some South Sandwich earthquakes (white diamonds). R-square value is 0.58.

**Figure 6:**
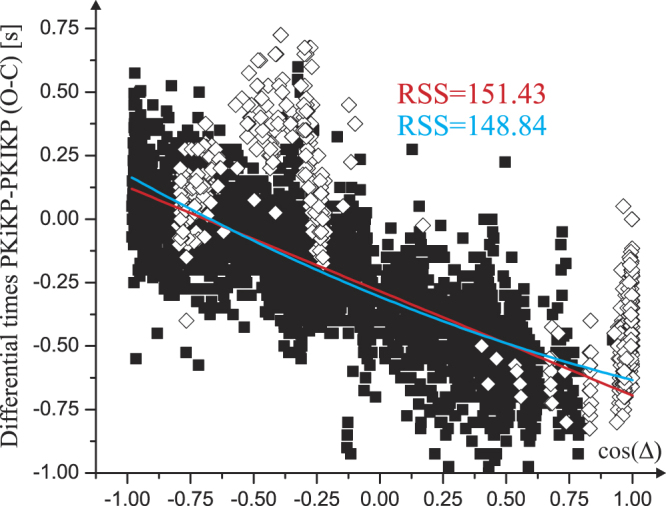
Differential times PKiKP-PKIKP vs. cosine of the angular distance of the PKIKP bouncing point to the (growth) G point (0, 80°W). The values of the Residual sum of Squares are indicated both for the linear (red, degree one spherical harmonic pattern, SHP) and the second order fit (blue, degree two SHP). The Akaike Criterion^42^ is minimized by the quadratic fit in respect to the linear fit.

We also investigated the possible degree one hemispherical pattern of the differential times considering the distance Δ to an equatorial pole located at 80°W^12^. Figure 5 shows the observations that provide limited support for such a model. The R-squared value obtained for a linear fit in Δ is increased by only 9% when the observations are fitted by a linear model in cos(Δ) (Fig. 6). According to a criterion from the information theory^42^, a better fit is represented by a degree two polynomial (in cos(Δ)). However, significant departures from a theoretical linear (or degree two) pattern are observed again near distances of 15° and 120°, especially due to some (but not all) of the South Sandwich events recordings.

## Conclusions

The spatial distribution of heterogeneity in the UIC shows important differences relative to the rest of the IC. It is commonly assumed that the former shows not only a quasi-hemispherical pattern, with a separation along certain meridians, but also anisotropy, with a north-south fast axis^43–45^.

The presence of two maxima (beneath Sumatra snd the south-eastern Africa) can be better explained by thermal models which allow a more complex pattern of the temperatures near ICB^16^. Our seismological observations provide important constraints to the mantle and core convection, leading to a better understanding of the Earth’s dynamo.

Neither the observed differential times nor the results of the inversion support a sharp boundary of the higher velocity cap, at least beneath the northern part of Eurasia.

There is a large variance of the amplitude ratio observations, suggesting a weak (if any) correlation between regions with higher velocity and lower attenuation in the UIC. It may support the possible presence of a mushy zone, or a mosaic-like ICB^46,47^. It may be also the effect of very short-scale heterogeneities (~75 km length) located near core-mantle boundary. The amplitude ratio of the high signal to noise recordings of the South Sandwich events observed at the Central Asia stations does not support models asking for a substantial change of the Q attenuation factor in the UIC in respect to ak135 model’ at least around (0°, 30° E)^48^.

## Electronic supplementary material

Supplementary Information
