# Supplementary material for: Non quasi-Hemispherical Seismological Pattern of the Earth’s Uppermost Inner Core
Source: Sci Rep. 2018 Feb 2;8:2270. doi: 10.1038/s41598-018-20657-x (PMC5797239; doi:10.1038/s41598-018-20657-x)
Supplement: Supplementary file 1 — Supplementary Information [file 41598_2018_20657_MOESM1_ESM.pdf]

Title: Non quasi-Hemispherical Seismological Pattern of the Earth's Uppermost Inner Core

Authors : Ivan, M., Wang, R., Hofstetter, R.

Supplementary Material 1: Testing the inversion on a chess-board model with two layers

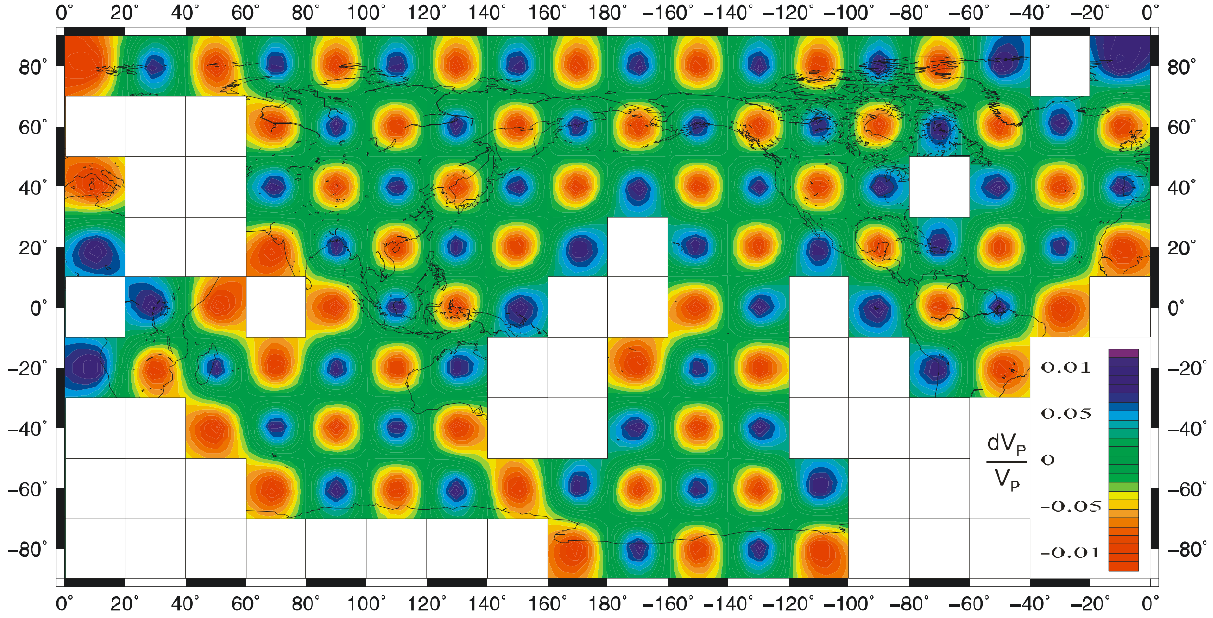

Figure S1. Results of the inversion for a checkerboard input model (first layer beneath ICB in the ak135 model). The velocity perturbation values are +/- 1.2 % for cells crossed by more than 43 rays and zero for the rest. The results explain more than 99% in the initial variance. White squares show the areas where the small number of rays did not allow reliable results. Figure produced with Generic Mapping Tools (GMT 5.1.2) [49].

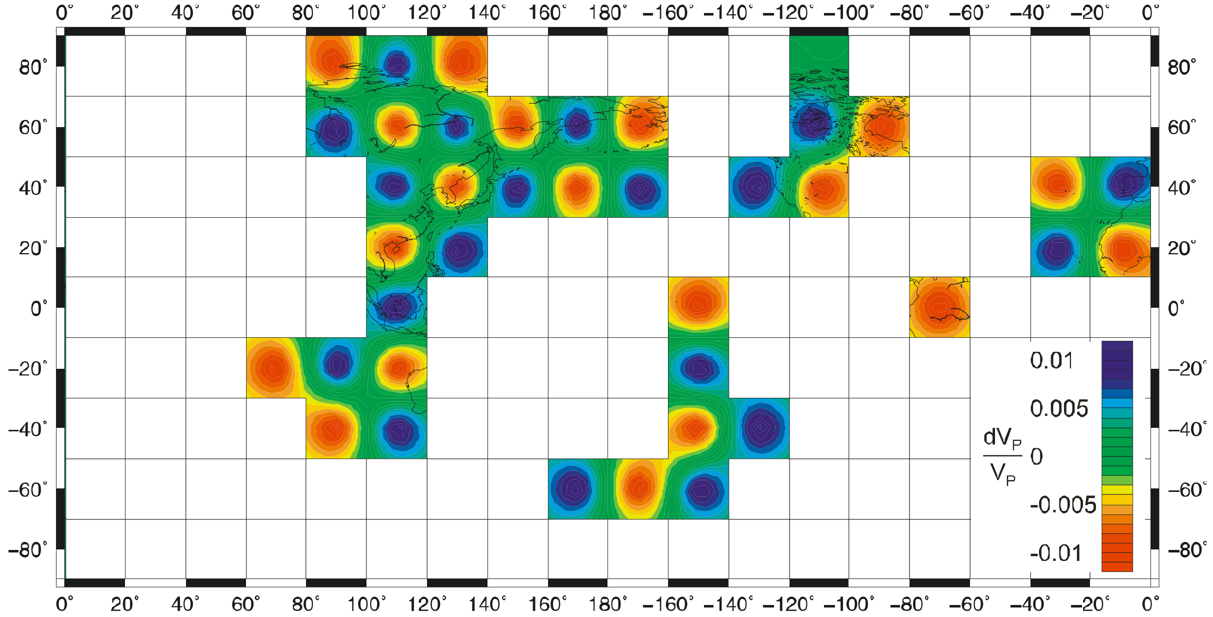

Figure S2. The same caption as in Figure S1, for the second layer beneath ICB in the ak135 model. Figure produced with Generic Mapping Tools (GMT 5.1.2) [49].

Title: Non quasi-Hemispherical Seismological Pattern of the Earth's Uppermost Inner Core

Authors : Ivan, M., Wang, R., Hofstetter, R.

Supplementary Material 2: Results of the inversion on real data

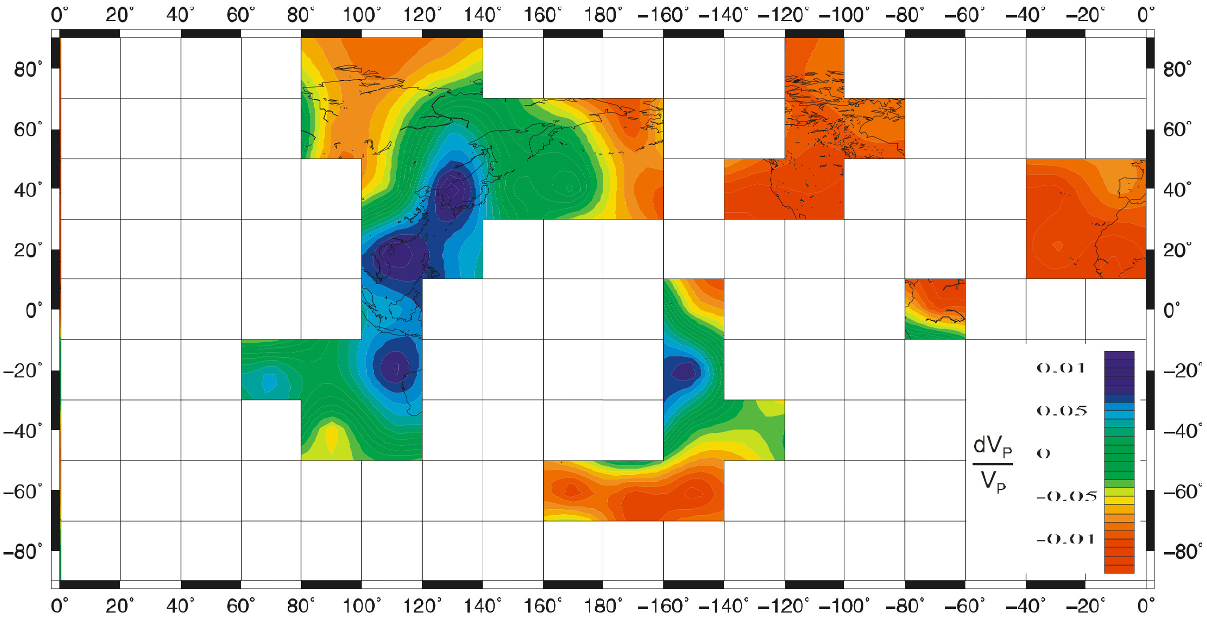

Figure S3. Velocity perturbations in the second layer beneath ICB in ak135 model (depths from 51.11 to 101.82 km beneath ICB). Same caption as in Figure 4. Figure produced with Generic Mapping Tools (GMT 5.1.2) [49].

Title: Non quasi-Hemispherical Seismological Pattern of the Earth's Uppermost Inner Core

Authors : Ivan, M., Wang, R., Hofstetter, R.

Supplementary Material 3: Gradual extinction (very low amplitudes) of PKiKP bouncing ICB around (0º, 70ºW)

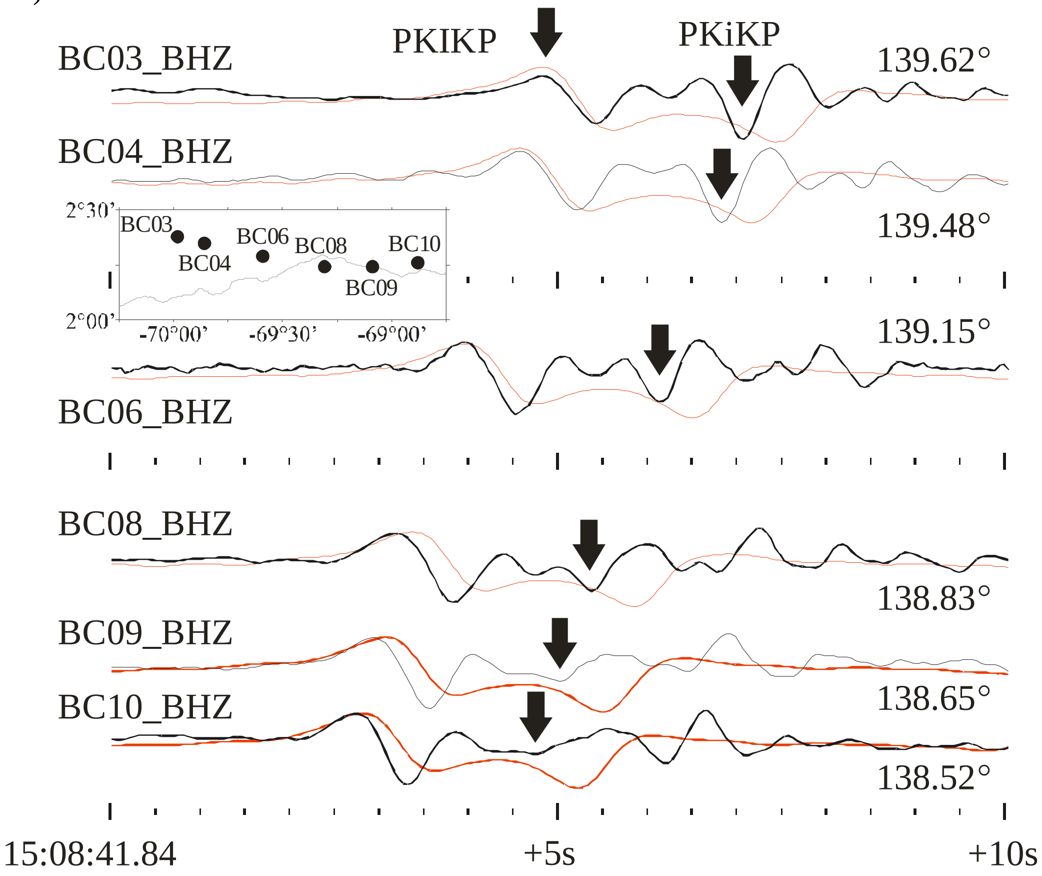

Figure S4. Raw broad-band recordings (black) at some Canadian stations (CANOE experiment) of the 2005, May, 4th South Sandwich event (Guralp CMG3ESP/Reftek instruments). All the corresponding synthetics (red) have been shifted with 0.8 seconds to align PKIKP arrival at BC06 station. Note the gradual decrease of PKiKP amplitude from BC03 to BC10. The inset shows the location of the stations.

Title: Non quasi-Hemispherical Seismological Pattern of the Earth's Uppermost Inner Core

Authors : Ivan, M., Wang, R., Hofstetter, R.

Supplementary Material 4: Testing the inversion on a chess-board model with one layer (101.82 km thick) beneath ICB and the inversion results

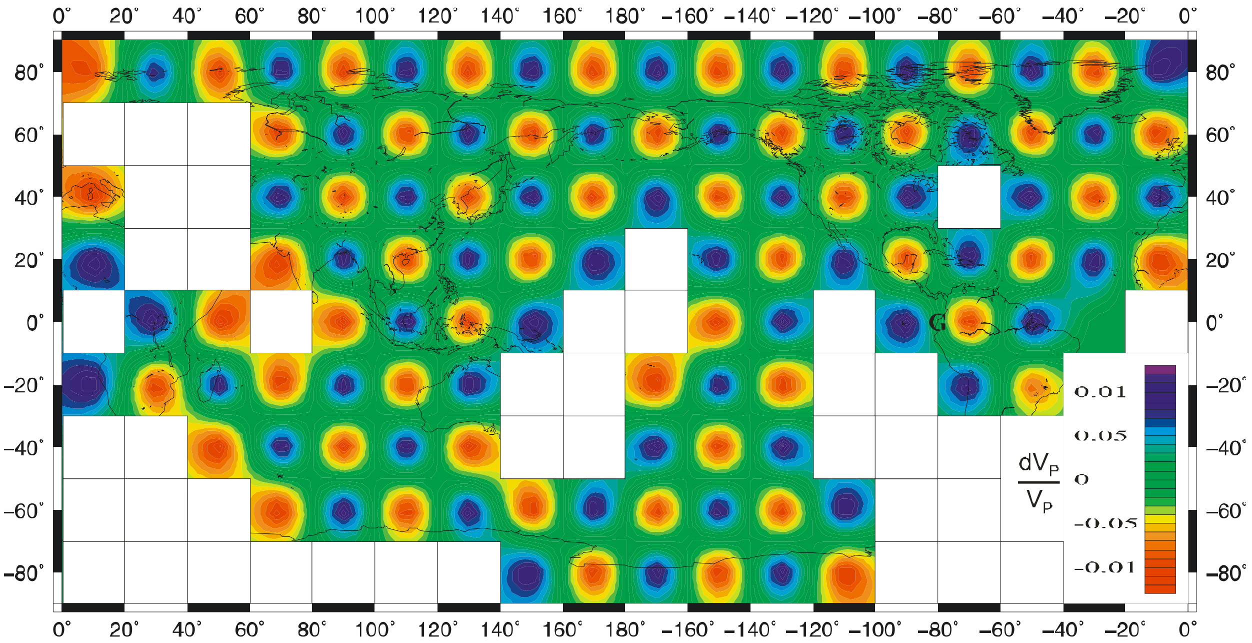

Figure S5. Results of the inversion for a checkerboard input model with a single layer (101.82 km thick) beneath ICB. The velocity perturbation values are +/- 1.2 % for cells crossed by more than 44 rays and zero for the rest. The results explain more than 99% in the initial variance. White squares show the areas where the small number of rays did not allow reliable results. Figure produced with Generic Mapping Tools (GMT 5.1.2) [49].

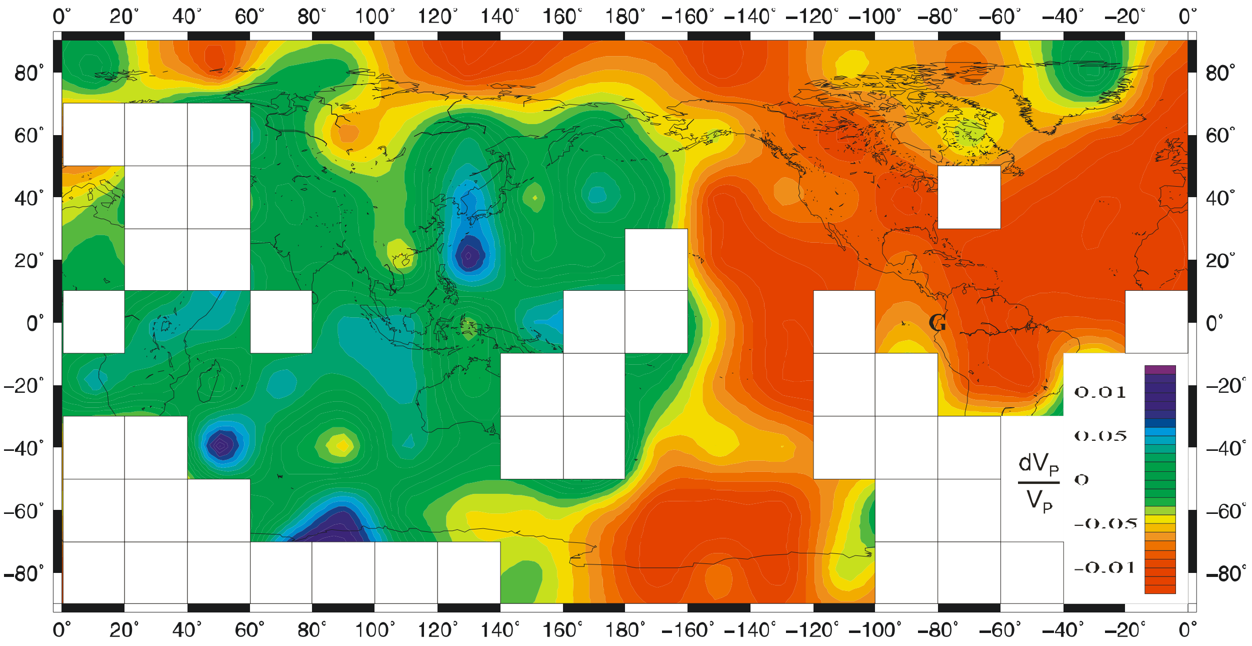

Figure S6. Velocity perturbation for the one layer model. The results explain more than 76% in the initial variance. Same caption as in Figure S5. Figure produced with Generic Mapping Tools (GMT 5.1.2) [49].

Title: Non quasi-Hemispherical Seismological Pattern of the Earth's Uppermost Inner Core

Authors : Ivan, M., Wang, R., Hofstetter, R.

Supplementary Material 5: Short description of the inversion

To a first order, the travel time perturbation δt is related to the relative velocity perturbation $\delta v / v$ by the following integral performed on the ray path in an undisturbed, 1-D model like ak135.

$$\delta t = - \int_{ray\_path} \delta v / v dt \quad (1)$$

Assuming the velocity perturbations are constant in each of the N cell crossed by the ray, the above integral is linearized as

$$\sum_{k}^{N} A_{ik} x_k = y_i \text{, } (2)$$

for all the differential times y_i. The travel time in a certain *k*-cell crossed by the *i*-th ray is denoted by A_{ik}, being evaluated analytically in the TauP approximation[1]. The unknown velocity perturbation in the *k*-cell is denoted by x_k. The inversion is done with an iterative, hyperplanar algorithm:

- assuming an initial ($iter = 0$) value of the perturbations, like $x_j^{iter=o} = 0$, then, for all the differential times
- $x_j^{iter+1} = x_j^{iter} + A_{ij} (y_i - \sum_{k}^{N} A_{ik} x_k^{iter}) / \sum_{k}^{N} A_{ik}^2 \quad (3)$

If x_j exceeds the upper (or the lower) limit previously imposed, it is set to the value of the upper (or lower) limit respectively. The variance is evaluated at each iteration. In the above examples, the perturbations had been limited in the range -1.2 to 1.2 % and the results had been obtained following 27 iterations (for the two layers model) and 13 iterations (for a single layer model). However, there is a very severe reduction in variance just beginning with the first iteration. For larger number of iterations, the algorithm started to provide only very small changes in the value of the variance at each step. And, due to accumulation of the round-off errors, it may begin to diverge slowly.

1. Crotwell, H. P., Owens, T. J., & Ritsema, J. The TauP toolkit: Flexible seismic travel-time and ray-path utilities. Seismological Research Letters, 70, 154_160 (1999).

Title: Non quasi-Hemispherical Seismological Pattern of the Earth's Uppermost Inner Core

Authors : Ivan, M., Wang, R., Hofstetter, R.

Supplementary Material 6: Table of events (NE = nuclear explosion)

Region	Date	OriginTime(UT)	Latitude	Longitude	Depth	Mag.
Santa_Cruz	1982-01-07	08:03:46.86	-12.1124	166.67090	176.9	5.7
Solomon	1987-06-18	14:03:18.34	-10.7217	162.27700	102.2	6.3
Leeward	1990-02-21	18:20:15.34	16.84980	-62.32650	119.3	5.9
N_Chile	1990-02-25	22:51:08.29	-18.2672	-69.12120	142.7	5.6
N_Peru	1990-03-13	19:40:34.95	-3.39160	-76.91080	122.6	5.6
NewZeeland	1990-03-22	00:00:16.65	-36.8506	177.14190	219.2	5.8

Tonga_Is	1990-05-20	07:32:39.45	-18.2049	-175.1969	258.8	6.3
FloresSea	1990-05-24	20:09:23.12	-7.36250	120.36430	589.1	6.4
Romania	1990-05-30	10:40:06.35	45.84740	26.662500	89.00	6.7
Fiji_Is	1990-06-24	08:35:25.77	-21.5851	-176.5039	202.1	5.5
Fiji_Is	1990-06-26	12:08:30.56	-21.9971	-179.5002	597.7	6.2
S_Fiji	1990-07-22	09:26:15.18	-23.5938	-179.9140	532.9	6.1
Near_Chile	1990-08-02	05:24:08.95	-31.6386	-71.70690	40.10	5.6
S_Sandwich	1990-08-06	20:30:43.07	-56.1260	-27.74450	93.90	5.6
Argentina	1990-09-04	23:15:10.58	-31.2191	-69.06170	103.7	5.5
Argentina	1990-09-07	16:09:19.97	-24.2582	-66.95840	165.4	5.4
Vanuatu	1990-09-28	19:44:50.40	-13.6087	167.04550	207.5	6.7
C_Peru	1991-04-09	06:02:25.17	-9.79700	-74.72040	130.1	5.8
S_Fiji	1991-04-18	09:41:20.36	-22.9074	-179.3607	472.4	6.0
Colombia	1992-01-02	19:41:46.38	5.669600	-73.83580	145.7	5.5
Vanuatu	1992-03-03	01:18:34.18	-14.4419	167.16800	176.7	5.7
S_Sandwich	1992-03-20	18:45:05.65	-56.4895	-27.26160	55.30	5.4
SWAtlantic	1992-06-22	04:00:41.31	-60.7590	-22.05020	11.80	6.3
Celebes	1992-07-12	23:41:00.51	3.093600	122.02720	628.5	5.7
N_Peru	1992-07-13	18:11:35.73	-3.88630	-76.58870	113.8	6.3
Colombia	1992-08-15	19:02:08.13	5.150700	-75.57450	106.6	5.9
S_Sandwich	1992-08-24	19:40:29.57	-56.6834	-26.81400	56.30	6.1
Fiji_Is	1992-08-30	20:09:06.31	-17.9194	-178.7136	571.8	6.4
Zaire_Cong	1992-09-11	03:57:26.54	-6.08080	26.639200	10.80	6.4
Vanuatu	1992-09-15	21:04:02.66	-14.1551	167.25520	211.5	6.2
Vanuatu	1992-12-14	07:41:01.06	-14.0466	170.75160	631.6	5.6
BandaSea	1992-12-23	03:00:42.99	-6.54460	130.44050	87.10	5.8
Tibet_Xiz	1993-03-20	14:51:59.67	29.02700	87.328400	12.20	6.2
S_Sandwich	1993-04-05	04:00:06.09	-59.7886	-26.27880	36.30	6.2
Fiji_Is	1993-04-20	16:26:18.88	-20.8321	-178.6795	581.0	5.8
W_Brazil	1993-05-06	13:03:20.27	-8.50470	-71.46480	600.6	6.1
Guerrero	1993-05-15	03:09:38.71	16.73850	-98.38640	19.70	6.0
Argentina	1993-06-08	23:17:41.04	-31.5157	-69.23600	107.8	6.3
Honduras	1993-06-12	11:15:07.89	13.25160	-87.54920	223.1	5.6
NETaiwan	1993-08-07	00:00:36.40	26.60630	125.67410	149.5	6.4
Guatemala	1993-08-22	10:02:11.42	14.23930	-90.69500	110.9	5.5
Solomon	1993-08-26	03:32:44.23	-5.48910	154.14630	153.0	5.7
Nicobar	1993-08-28	20:14:43.03	6.496600	94.650300	110.3	5.9
S.Sumatera	1993-09-01	11:48:40.08	-4.32990	102.59130	86.40	5.7
HinduKush	1993-09-04	11:38:39.99	36.40790	70.816200	204.8	6.0
S_Sandwich	1993-09-09	21:52:08.44	-56.2160	-27.32000	69.60	5.6
Mexico_Chi	1993-09-10	18:58:56.73	14.40660	-92.78980	60.60	6.0
Solomon_Is	1993-09-22	12:37:08.63	-6.47460	154.94480	67.10	6.1
NewBritain	1993-09-29	19:03:09.06	-6.02500	149.36340	82.40	5.9
LopNor_NE	1993-10-05	01:59:56.68	41.63220	88.688600	0.000	5.9
PapuaNG	1993-11-10	00:03:25.42	-4.69510	151.92050	113.4	5.4
S_Sandwich	1993-12-01	00:59:02.98	-57.4872	-25.89810	44.60	5.7
Fiji_Is	1993-12-24	05:18:34.35	-21.8702	-178.6385	443.8	5.8
N_Peru	1994-01-20	09:06:52.25	-6.02520	-77.06330	118.6	5.7
Tonga_Is	1994-02-24	15:25:29.56	-17.3574	-174.2718	70.10	6.1
Fiji_Is	1994-04-18	21:39.42.74	-21.4639	-178.8010	539.1	5.9
Fiji_Is	1994-04-20	23:35:30.70	-17.9293	-178.3868	552.5	5.9
Vanuatu	1994-04-23	15:00:54.03	-14.1875	167.55770	18.20	6.1
S.Sumatera	1994-05-02	17:14:03.94	-1.10170	97.544000	37.50	6.1
Vanuatu	1994-05-04	06:37:37.88	-17.0950	168.27400	221.3	6.2
BandaSea	1994-06-16	10:52:50.63	-7.40730	128.16950	141.9	5.9
S_Peru	1994-06-16	18:41:28.72	-15.3356	-70.29010	207.0	5.9
Afghanistan	1994-06-30	09:23:22.22	36.40510	71.157500	232.1	6.3
Fiji_Is	1994-07-05	02:59:43.47	-16.3788	-177.4519	425.3	5.9
BandaSea	1994-07-13	11:45:22.39	-7.50340	127.84630	150.4	6.4
SSandwich	1994-07-25	04:39:18.10	-57.0041	-25.57530	33.00	5.6
Peru_Boliv	1994-08-08	07:55:40.14	-13.8472	-68.33210	609.7	5.6
Myanmar	1994-08-08	21:08:31.09	24.7115	95.212300	116.1	6.1
SSandwich	1994-08-10	14:57:49.64	-58.7575	-25.74850	33.00	5.5
Fiji_Is	1994-08-11	19:32:52.13	-21.9636	-176.6695	182.5	5.9
Argentina	1994-08-19	10:02:51.61	-26.6004	-63.38180	558.3	6.5
Lake_Baykal	1994-08-21	15:55:59.32	56.74790	117.91950	12.60	6.0

Santa_Cruz	1994-08-22	17:26:39.67	-11.5935	166.44810	164.3	6.2
JavaSea	1994-09-28	17:34:00.24	-5.76770	110.50280	660.2	6.0
Fiji_Is	1994-09-30	19:30:16.29	-21.2715	-179.1884	615.2	5.8
HinduKush	1994-10-25	00:54:34.44	36.39470	70.991900	238.3	6.0
Santa_Cruz	1994-11-03	03:07:23.69	-11.0371	166.23670	178.3	5.8
Peru_Brazil	1994-11-04	01:13:22.18	-9.40760	-71.30440	617.7	6.1
Peru_Brazil	1994-11-05	12:05:29.29	-9.38560	-71.32820	605.4	5.6
S_Peru	1994-11-11	08:48:29.91	-15.6369	-72.56390	121.8	5.8
JavaSea	1994-11-15	20:18:11.46	-5.61770	110.25940	567.7	6.5
Fiji_Is	1994-12-18	20:38:30.98	-17.8727	-178.6585	532.3	5.7
Kermadec	1994-12-27	17:32:51.86	-32.0525	179.85790	220.5	6.4
S_Sandwich	1995-01-03	02:54:54.27	-56.2007	-27.39050	105.1	5.2
Drake_Pass.	1995-01-03	16:11:57.08	-57.6794	-65.96700	13.90	5.9
Fiji_Is	1995-01-17	16:54:11.00	-20.8588	-179.2129	622.7	6.3
BandaSea	1995-01-19	09:55:35.16	-7.40630	128.35160	173.7	5.9
E_NewGuinea	1995-03-12	12:09:44.76	-5.38270	146.70950	242.1	6.0
Mariana	1995-04-08	17:45:16.40	21.80170	142.68970	301.8	6.2
Vanuatu	1995-04-13	02:34:36.60	-13.5152	170.48900	623.0	6.2
Vanuatu	1995-05-05	22:48:06.00	-18.5517	168.74600	127.0	5.9
Myanmar	1995-05-06	01:59:07.04	24.96050	95.294900	117.6	6.4
JavaSea	1995-05-13	21:00:56.86	-5.31870	108.95600	574.4	5.9
HinduKush	1995-05-16	03:35:03.60	36.45540	70.908900	195.9	5.8
ScotiaSea	1995-05-30	16:56:25.23	-60.2473	-31.62870	33.00	5.6
NewIreland	1995-06-24	06:58:08.41	-3.95680	153.91280	403.8	6.8
Chile_Arg	1995-08-03	08:18:52.36	-28.2137	-69.21230	89.70	5.8
Afghanistan	1995-08-17	23:14:19.90	36.45330	71.142400	241.4	5.6
S_Sandwich	1995-08-18	02:16:26.77	-55.7915	-28.84180	37.90	6.2
Colombia	1995-08-19	21:43:30.71	5.183600	-75.57200	105.6	6.6
Mariana	1995-08-24	01:55:36.45	18.82780	145.05460	612.0	6.2
Fiji_Is	1995-09-14	12:24:32.97	-17.7070	-178.9289	517.8	5.8
Vanuatu	1995-09-26	18:24:14.81	-13.1830	166.99700	204.9	5.8
S_Fiji	1995-10-14	08:00:53.38	-25.7544	-177.5571	175.7	6.2
Vanuatu	1995-10-23	03:58:09.43	-14.3455	167.29730	207.0	5.9
Fiji_Is	1995-12-10	23:46:59.79	-21.4973	-178.0574	409.2	6.2
Tuamotu_NE	1996-01-27	21:29:58.16	-22.1654	-138.8348	0.000	5.2
Molucca	1996-02-28	09:44:09.34	1.717700	126.13480	107.1	6.4
Kuril_Is	1996-05-07	23:19:58.70	43.62950	147.65990	37.50	6.2
S_Sandwich	1996-05-23	03:38:39.89	-55.9949	-27.83400	111.4	5.3
S_Sandwich	1996-05-30	03:04:32.89	-56.7111	-26.44280	38.90	6.0
Vanuatu	1996-08-11	01:31:11.10	-13.4412	166.82960	49.10	6.0
Vanuatu	1996-08-15	07:33:52.67	-13.3226	166.84540	45.10	6.2
BandaSea	1996-08-22	05:35:43.16	-7.15990	123.35020	614.0	5.9
Fiji_Is	1996-08-27	06:24:06.69	-22.5295	-179.7499	559.0	5.9
Santa_Cruz	1996-09-01	06:45:41.08	-11.8610	166.65180	183.9	5.8
NearHonshu	1996-09-11	02:37:15.30	35.58920	141.01770	54.20	6.2
Nicaragua	1996-09-18	17:34:24.95	11.45790	-85.53690	233.6	5.6
Leeward	1996-09-24	11:42:19.14	15.20260	-61.45070	148.1	5.8
Fiji_Is	1996-11-14	13:47:38.69	-21.3883	-176.5184	202.9	6.2
Nicaragua	1996-11-17	19:17:49.70	11.11970	-86.13460	103.1	6.0
PapuaNG	1996-12-26	19:34:10.57	-5.41500	151.45530	98.70	5.8
Chiapas	1996-12-31	12:41:40.93	15.77790	-93.04230	90.00	6.4
Minahassa	1997-01-01	22:32:35.40	-0.12420	123.87400	124.6	5.8
Solomon	1997-02-08	01:55:55.22	-8.54350	158.98590	96.70	5.7
S_Sandwich	1997-02-14	23:43:44.00	-56.4466	-27.51420	144.5	5.4
Fiji_Is	1997-03-11	03:13:55.99	-21.0936	-178.7692	507.1	5.7
Kermadec	1997-03-21	12:07:16.20	-31.2645	179.65330	434.5	6.3
Peru_Brazil	1997-03-25	16:44:34.02	-9.05110	-71.24800	619.2	6.0
N.Chile	1997-04-01	18:33:32.34	-18.2548	-69.47510	113.7	6.2
S_Sandwich	1997-04-02	03:04:41.66	-58.2751	-25.50970	35.80	5.0
Kermadec	1997-04-12	09:21:47.33	-28.0954	-178.3150	98.50	6.0
Vanuatu	1997-04-27	00:31:34.2	-19.1169	168.6796	51.10	6.0
N_Chile	1997-05-17	02:10:15.89	-27.1000	-69.47730	77.40	5.5
S_India	1997-05-21	22:51:28.09	23.09110	80.081800	29.10	5.8
Mexico	1997-05-22	07:50:55.31	18.67060	-101.6584	84.60	6.5
S_Sandwich	1997-05-29	00:12:32.96	-55.7705	-27.13260	33.00	6.1
S_Sandwich	1997-06-02	21:24:39.04	-57.8622	-25.64200	56.80	5.4

E.NewGuinea	1997-06-12	12:07:37.61	-6.00570	147.00010	67.10	6.1
S_Sandwich	1997-06-15	13:01:10.98	-56.9045	-24.96380	33.00	5.4
ElSalvador	1997-08-24	00:59:42.94	13.30360	-89.59360	71.60	5.6
S_Fiji	1997-08-26	15:22:08.09	-25.5398	178.36600	596.9	5.7
PapuaNG	1997-09-04	20:53:01.70	-3.53170	151.42140	400.0	5.7
Solomon	1997-09-07	12:57:06.27	-6.02310	154.48530	415.6	5.8
BandaSea	1997-09-26	15:48:34.43	-5.36690	128.98440	253.6	6.0
N_Chile	1997-09-28	23:13:13.80	-22.4376	-68.41950	107.3	5.6
S_Sandwich	1997-10-05	18:04:31.14	-59.7372	-29.43930	274.3	6.3
Kermadec	1997-10-08	10:47:49.42	-29.1533	178.35090	606.9	5.7
Kuril_Is	1997-10-22	09:55:48.78	44.68000	146.27150	163.3	5.6
Vanuatu	1997-10-31	12:29:52.72	-13.1534	166.89050	206.8	5.9
Banda_Sea	1997-11-03	19:17:59.44	-6.72430	129.03420	220.9	6.1
Nicaragua	1997-11-06	17:29:08.45	11.71930	-85.81330	119.3	5.6
ElSalvador	1997-11-09	22:56:43.86	13.88970	-88.80160	183.1	6.4
Hokkaido	1997-11-15	07:05:16.32	43.79310	145.03450	158.0	6.1
Kermadec	1997-11-18	15:41:27.66	-29.1979	-177.5394	37.80	5.8
S_Sandwich	1997-11-20	06:08:10.96	-59.0682	-25.55820	33.00	5.5
Fiji_Is	1997-11-29	02:42:27.01	-20.9922	-178.7501	572.5	5.7
Colombia	1997-12-11	07:56:29.66	3.972900	-75.76530	182.4	6.4
ElSalvador	1997-12-18	15:02:00.72	13.83080	-88.75560	186.0	6.0
Guatemala	1998-01-10	08:20:10.12	14.4023	-91.57390	70.50	6.6
S_Sandwich	1998-01-13	08:49:14.33	-55.5753	-28.29330	41.90	5.5
S_Fiji	1998-01-26	18:30:25.92	-22.1488	-176.7390	115.4	5.7
Fiji_Is	1998-01-27	02:14:10.78	-20.8330	-179.0944	616.6	5.9
S_Fiji	1998-01-27	21:05:42.75	-22.4634	179.12300	588.1	6.5
S_Sandwich	1998-02-06	13:01:15.55	-56.1077	-27.76000	119.0	5.4
Vanuatu	1998-02-07	03:20:18.58	-14.8061	167.33680	126.8	5.9
Vanuatu	1998-02-28	10:46:54.41	-14.4817	167.32860	204.6	5.8
Guatemala	1998-03-03	02:24:45.05	14.38500	-91.47970	73.10	6.1
Argentina	1998-03-25	21:02:52.42	-24.2160	-66.88760	164.7	5.6
S_Kermadec	1998-04-10	16:43:40.50	-33.5361	-179.5551	35.80	5.9
S_Fiji	1998-04-14	03:41:19.96	-23.7743	-179.8343	468.8	6.1
JawaSea	1998-04-27	23:51:36.05	-6.09050	113.14200	598.6	5.7
Fiji_Is	1998-04-28	15:44:04.32	-21.9938	-179.4874	581.5	5.7
Fiji_Is	1998-05-16	10:41:23.42	-21.7224	-176.6263	125.1	5.5
S_Sandwich	1998-05-20	06:17:51.92	-56.1456	-27.55520	95.70	4.7
Mindanao	1998-05-23	17:44:46.74	8.177800	123.78850	646.0	6.0
SanJuan	1998-06-07	16:10:45.33	-31.4700	-67.78630	103.0	5.9
S_Fiji	1998-06-12	20:50:58.43	-24.7011	179.85180	460.5	5.5
Vanuatu	1998-07-25	02:39:25.69	-13.6357	166.83620	59.70	6.3
S_Sandwich	1998-07-30	23:36:30.89	-58.8092	-25.29410	33.00	5.4
Luzon_Phil	1998-08-23	05:36:13.22	14.72250	120.05090	73.00	6.0
S_Molucca	1998-08-28	12:40:58.56	-0.15450	125.08160	73.00	6.1
Tonga_Is	1998-09-01	01:19:33.74	-17.4887	-174.7576	182.8	5.6
S_Sandwich	1998-09-01	10:29:48.48	-58.2387	-26.61530	143.0	5.6
Minahassa	1998-09-21	06:52:44.65	0.194200	122.46860	181.3	5.9
Ryukyu_Is	1998-10-03	11:15:41.68	28.52640	127.64890	216.5	6.1
S_Peru	1998-10-08	04:51:40.41	-16.0485	-71.34720	111.6	6.2
Fiji_Is	1998-10-11	12:04:52.29	-21.0315	-179.0495	592.5	5.9
Argentina	1998-10-11	21:44:16.36	-27.2931	-63.31410	582.3	5.4
Tonga_Is	1998-11-24	23:54:44.10	-16.4966	-174.7150	204.2	6.0
W_Brazil	1998-12-10	08:21:12.77	-7.92190	-71.32500	624.4	5.5
Vanuatu	1999-01-13	20:12:17.43	-20.6010	169.71400	140.8	5.7
Fiji_Is	1999-01-25	10:37:10.39	-17.9390	-178.4270	598.9	5.8
PapuaNG	1999-01-28	18:24:25.98	-4.62300	153.63500	109.2	6.4
Santa_Cruz	1999-02-05	11:39:46.54	-12.6520	166.97000	224.5	5.9
N_Chile	1999-03-02	17:45:54.89	-22.8020	-68.49100	110.2	5.9
Chile_Bol	1999-03-05	00:33:41.94	-20.3370	-68.83700	110.9	5.8
Fiji_Is	1999-03-06	20:28:51.61	-21.6920	-179.3980	571.9	5.7
New_Britain	1999-04-11	16:50:40.32	-6.01100	148.42200	70.70	6.0
Ecuador	1999-04-26	18:17:27.76	-1.64200	-77.73300	185.3	6.0
NewZeeland	1999-05-18	09:19:33.79	-38.6280	175.19400	272.7	5.7
Argentina	1999-05-25	16:42:02.35	-27.7890	-66.73600	137.3	5.8
S_Sandwich	1999-06-22	18:48:17.15	-56.2610	-27.64300	85.10	5.5
Fiji_Is	1999-06-26	22:05:29.09	-17.9370	-178.1940	585.5	6.0

Kuril_Is	1999-07-07	18:52:58.01	49.20400	155.55100	41.30	6.1
Solomon	1999-07-09	05:04:48.43	-6.59300	154.87600	77.50	6.3
Fiji_Is	1999-07-21	03:10:43.80	-18.2740	-177.9050	549.3	5.7
Kermadec	1999-07-28	00:16:57.90	-28.7330	-177.5230	36.60	6.0
Kermadec	1999-07-28	10:08:23.35	-30.3420	-178.0670	47.10	6.3
Ecuador	1999-08-03	15:58:58.96	-3.41400	-79.15100	98.40	5.9
Vanuatu	1999-09-18	23:51:32.53	-19.7370	169.21300	117.3	5.9
N.Sumatera	1999-11-11	18:05:44.02	1.246000	100.21400	214.6	6.2
Chile_Bol	1999-11-21	03:51:14.50	-21.7040	-68.76000	100.7	5.9
C_Peru	1999-11-27	02:31:46.41	-14.4650	-71.27800	96.70	5.9
Fiji_Is	1999-11-30	20:10:19.18	-21.2390	-178.6190	503.9	5.8
Tonga_is	1999-12-07	21:29:42.45	-15.9520	-173.9170	77.40	6.4
Guerrero	1999-12-29	05:19:46.91	18.24000	-101.4950	66.70	5.9
Fiji_Is	2000-01-13	20:07:12.07	-17.5500	-178.7140	501.6	6.2
Taiwan	2000-01-28	16:39:23.19	26.06200	124.54700	184.7	6.0
Mariana	2000-02-15	02:05:03.60	17.62200	145.44800	555.8	5.8
Fiji_Is	2000-03-01	04:21:01:28	-19.2000	-179.2740	679.4	5.8
Tonga_Is	2000-04-07	18:42:21.10	-18.2810	-175.2160	184.0	5.7
Kermadec	2000-04-11	06:41:25.71	-28.1620	-178.3200	201.2	5.8
Fiji_Is	2000-04-18	17:28:08.31	-20.5900	-176.4370	182.3	6.0
Argentina	2000-04-23	17:01:17.29	-28.3320	-62.92600	604.6	6.1
NewBritain	2000-05-08	10:28:25.64	-4.45700	150.01700	503.4	6.1
Kermadec	2000-05-08	21:35:42.04	-31.2940	179.85500	370.2	5.6
S_Fiji	2000-06-14	02:15:28.01	-25.6260	178.05800	631.2	6.4
Kermadec	2000-06-16	20:23:32.77	-29.2570	-178.2770	224.0	5.6
S_Sumatera	2000-07-10	10:39:39.15	-4.50100	103.76600	106.7	5.8
BandaSea	2000-08-07	14:33:56.81	-6.97900	123.42500	666.1	6.5
Kermadec	2000-08-15	04:30:08.23	-31.5610	179.74400	349.6	6.6
Fiji_Is	2000-09-02	10:19:10.61	-17.8470	-178.2980	550.6	5.7
Fiji_Is	2000-09-02	17:02:17.15	-19.9530	-179.0840	650.3	6.0
S_Sandwich	2000-09-11	10:03:14.17	-57.7520	-25.27500	39.60	5.3
Halmahera	2000-09-26	16:49:35.16	1.122000	127.52900	160.3	5.9
Mexico	2000-10-17	20:00:37.17	15.65800	-92.03800	182.2	5.8
Peru-Brazil	2000-11-01	04:27:44.96	-7.96900	-74.35400	144.5	5.9
Fiji_Is	2000-12-18	01:19:21.31	-21.1540	-179.1150	617.7	6.5
PapuaNG	2000-12-21	01:01:34.93	-5.84900	151.13200	96.40	6.3
Solomon	2000-12-21	02:41:23.80	-5.38500	154.16500	391.9	6.0
Kuril_Is	2001-02-07	15:16:15.65	52.70700	153.84100	432.1	5.7
Bali_Sea	2001-02-16	05:59:09.60	-7.13800	117.48700	526.6	6.0
S_Fiji	2001-03-11	00:50:40.20	-25.5400	-177.9540	230.7	5.8
Argentina	2001-04-21	18:15:46.0	-29.0670	-67.46100	119.8	5.7
Fiji_Is	2001-05-19	17:36:26.60	-19.9360	-177.4920	374.7	5.9
N_Chile	2001-06-18	19:56:56.22	-24.3400	-69.21200	88.6d	5.8
Chile_Bol	2001-06-19	09:32:24.18	-22.8290	-67.97500	145.0	5.9
S_Bolivia	2001-06-29	18:35:50.94	-19.7250	-66.27200	271.9	6.1
Fiji_Is	2001-07-04	07:06:30.93	-21.7350	-176.7270	178.7	6.5
Tonga_Is	2001-07-19	20:12:13.59	-17.0380	-174.5780	210.1	5.8
NewBritain	2001-08-09	18:57:50.38	-5.54000	149.64400	151.6	5.4
S_Fiji	2001-09-26	21:31:12.48	-26.6350	178.26500	634.9	5.8
S_Sandwich	2001-10-01	04:18:13.38	-58.3160	-25.57100	18.20	5.6
Vanuatu	2001-10-26	23:05:50.35	-18.5140	168.14700	26.00	6.1
Fiji_Is	2001-11-05	23:07:10.89	-17.3390	-179.1560	551.6	6.2
Mexico	2001-11-28	14:32:33.68	15.67900	-93.12900	86.20	6.4
Vanuatu	2001-12-12	12:53:20.39	-17.2060	167.65300	50.30	6.1
Hindu Kush	2002-01-03	07:05:26.10	36.03900	70.679000	115.0	6.0
Mariana	2002-01-07	13:26:29.34	18.93300	144.97100	633.8	5.9
Kuril_Isls	2002-01-28	13:50:31.13	49.37600	155.61300	52.70	6.1
New_Britain	2002-01-30	12:58:20.01	-6.25000	150.89000	41.60	6.0
S_Sandwich	2002-02-10	01:47:07.45	-55.9750	-29.15200	198.0	5.9
PapuaNG	2002-02-23	19:37:15.13	-4.47500	152.02900	167.4	5.5
S_Sandwich	2002-03-09	12:27:11.64	-56.1230	-27.49200	116.1	6.0
S_Sumatera	2002-06-16	18:31:09.73	-2.35900	102.47200	220.3	5.7
Fiji_Is	2002-06-16	06:55:13.39	-17.9200	-178.6500	571.2	5.9
Tonga_Is	2002-06-21	14:21:41.86	-15.1480	-175.8220	290.3	5.7
Santa_Cruz	2002-06-29	02:39:04.68	-12.4800	166.54100	66.00	6.1
S_Fiji	2002-06-30	21:29:36.65	-22.2370	179.23900	626.5	6.4

Fiji_Is	2002-08-09	13:31:04.00	-16.3790	-176.1190	350.5	6.1
Argentina	2002-09-24	03:57:20.90	-31.4490	-69.13900	104.6	6.2
BaliSea	2002-10-03	19:05:11.63	-7.51340	115.67660	325.8	6.0
Fiji_Is	2002-10-22	11:39:04.88	-20.6722	-178.4125	555.4	6.1
Nicobar	2002-10-24	21:53:42.99	6.035200	94.426700	63.60	5.7
S_Sandwich	2002-11-12	01:46:49.74	-56.5818	-27.71680	118.8	6.2
S_Fiji	2002-12-10	04:27:54.79	-24.1952	179.2548	531.4	6.0
Chiapas	2003-02-01	16:30:58.42	16.73830	-93.02190	211.4	5.8
S_Fiji	2003-02-21	22:13:21.11	-26.1959	-178.2239	222.4	5.8
Vanuatu	2003-04-05	22:03:31.98	-16.1975	167.88240	175.7	5.8
N_Halmahera	2003-05-05	23:04:45.98	3.644700	128.00990	61.60	6.1
Kermadec	2003-06-05	08:23:16.58	-31.1134	-178.6866	117.4	5.7
Solomon	2003-06-14	18:28:48.73	-7.59190	156.77960	396.6	5.7
PapuaNG	2003-07-21	13:53:59.87	-5.50180	148.80660	196.6	6.3
ScotiaSea	2003-08-04	18:18:30.19	-60.5817	-43.13320	10.00	5.3
Andaman	2003-08-11	21:22:30.44	12.12490	93.497100	100.3	6.0
Guatemala	2003-08-25	06:28:34.96	13.99320	-91.12550	99.50	5.9
BandaSea	2003-08-28	06:38:13.88	-7.30690	126.06490	434.2	5.9
Primorye	2003-08-31	23:07:59.16	43.39710	132.31600	467.3	6.1
N_Sumatera	2003-09-05	01:23:02.64	5.301000	95.899100	131.6	5.9
S_Sandwich	2003-09-06	15:47:03.18	-57.4636	-25.58210	57.30	5.7
New_Britain	2003-09-12	06:55:56.40	-5.26590	151.50010	51.40	6.0
Chile_Bol	2003-09-17	21:34:47.29	-21.4270	-68.27000	126.1	5.7
S_Sandwich	2003-10-20	16:50:17.20	-58.0868	-26.25060	140.8	5.5
Volcano	2003-11-11	18:48:25.04	22.37390	143.36970	108.9	5.9
Kyrgyzstan	2003-12-01	01:38:35.39	42.88090	80.523300	33.00	6.0
Fiji_Is	2004-01-11	08:07:03.70	-16.3485	-176.1014	366.0	5.9
Tonga_Is	2004-01-25	11:43:10.38	-16.8453	-174.1696	129.6	6.6
Argentina	2004-02-04	05:18:43.49	-26.1088	-63.42340	551.9	5.7
S_Sumatra	2004-02-22	06:46:26.87	-1.58020	100.41260	51.30	6.0
BandaSea	2004-04-17	15:58:26.47	-7.42740	128.38650	158.3	5.8
Costa_Rica	2004-04-29	00:57:25.09	10.72050	-86.11150	53.2	6.2
S_Sandwich	2004-05-05	20:59:38.43	-57.2272	-25.53210	42.00	5.5
S_Sandwich	2004-05-07	09:50:27.39	-57.8706	-25.62620	45.70	5.4
Java_Sea	2004-06-06	09:38:05.90	-6.05110	113.11260	576.9	5.8
Santa_Cruz	2004-06-22	09:04:41.34	-10.9349	166.20610	143.0	5.8
Mexico	2004-08-07	11:49:11.71	17.32220	-95.26590	99.80	5.8
Santa_Cruz	2004-08-12	15:59:40.57	-11.9552	166.67710	205.7	5.7
S_Sandwich	2004-10-08	15:28:37.39	-56.5168	-26.88620	95.00	5.8
S_Fiji	2004-11-23	21:04:56.62	-24.3776	179.01980	549.9	5.8
BismarckSea	2004-12-11	01:55:48.29	-4.61590	149.75710	531.9	5.5
Mindanao	2005-01-21	17:54:33.71	5.051200	125.24780	178.0	5.8
S_Sandwich	2005-01-31	13:56:36.66	-56.0659	-27.42800	114.2	5.5
N_Peru	2005-04-11	14:54:05.41	-7.31360	-77.96200	128.6	6.0
Peru_Bol	2005-04-16	22:41:14.65	-17.6207	-69.70360	118.8	5.8
S_Sandwich	2005-05-04	14:49:25.65	-57.9438	-25.65990	65.00	5.3
S_Sandwich	2005-05-18	09:10:51.08	-56.4069	-26.76840	84.80	6.0
S_Sandwich	2005-06-12	19:26:23.94	-56.3234	-27.19220	91.60	6.0
Vanuatu	2005-07-23	01:04:24.87	-15.1969	167.46960	126.9	5.5
S_Sandwich	2005-08-04	12:11:18.94	-59.6285	-25.84590	44.30	5.4
N_Chile	2005-08-14	02:39:39.18	-19.7422	-69.08090	114.6	5.8
S_Sandwich	2005-09-09	01:20:16.86	-55.6422	-27.12310	10.00	5.7
Argentina	2005-09-09	11:26:05.64	-31.6236	-69.14110	114.0	5.7
S_Sandwich	2005-09-09	19:55:18.63	-56.0067	-27.94670	125.0	5.6
Fiji_Is	2005-09-12	21:15:04.24	-17.4748	-177.3684	402.7	5.7
Taiwan	2005-10-15	15:51:07.57	25.29650	123.43520	200.4	6.4
JapanSea	2005-10-23	10:08:14.19	37.43690	134.57790	383.4	5.9
NewGuinea	2005-10-25	19:40:42.29	-7.12870	145.94020	178.9	5.8
Ecuador	2005-11-09	11:33:11.80	-1.04180	-76.98420	247.6	5.9
Chile_Bol	2005-11-17	19:26:54.8	-22.3676	-67.94210	161.7	6.8
Afghanistan	2005-12-12	21:47:44.65	36.40590	71.103500	223.9	6.5
Fiji_Is	2006-02-02	12:48:43.88	-17.8306	-178.2842	599.6	6.7
S_Sandwich	2006-04-30	03:51:29.26	-59.6954	-26.16140	32.40	5.3
S_Sandwich	2006-05-13	23:53:31.12	-56.2159	-27.71470	94.60	5.1
Kamchatka	2006-05-22	13:08:02.16	54.33240	158.41410	190.1	6.2
Fiji_Is	2006-06-27	02:59:16.49	-19.9576	-178.2300	574.9	6.3

BandaSea	2006-07-15	07:10:49.00	-4.48140	126.25820	370.5	5.8
Argentina	2006-09-22	02:32:24.60	-26.8454	-63.11470	583.0	6.0
Solomon	2006-12-27	20:15:40.93	-5.78080	154.45800	373.5	6.0
Vanuatu	2007-01-23	17:16:21.30	-13.1260	167.02690	189.3	5.9
NewIreland	2007-04-21	07:12:48.32	-3.55370	151.35110	407.8	6.1
Fiji_Is	2007-05-06	21:11:53.31	-19.4681	-179.3288	678.6	6.5
Fiji_is	2007-05-13	11:26:43.74	-19.5716	-179.3121	672.2	5.8
Argentina	2007-05-25	17:47:31.80	-24.2493	-66.93940	181.7	5.9
Russia	2007-05-30	20:22:13.56	52.15230	157.21670	122.3	6.4
S_Sandwich	2007-06-14	06:58:13.21	-56.4303	-27.30310	137.9	5.2
S_Sandwich	2007-06-15	03:48:35.53	-58.6013	-26.21830	150.4	5.5
Mexico	2007-07-06	01:09:20.36	16.61690	-93.54130	121.9	6.0
W_Brazil	2007-07-21	13:27:03.95	-8.08590	-71.20710	633.7	6.0
S_Sandwich	2007-07-31	02:42:49.58	-56.1028	-27.73180	113.3	5.7
Ecuador	2007-09-26	04:43:18.01	-3.95800	-79.21740	102.1	5.9
S_Fiji	2007-10-05	07:17:54.26	-25.1987	179.44620	521.3	6.5
S_Fiji	2007-10-16	21:05:43.17	-25.7449	179.50190	501.2	6.6
Argentina	2007-11-18	05:40:09.05	-22.6479	-66.26550	216.4	6.0
Fiji_Is	2007-11-19	00:52:12.68	-21.2118	-178.6727	558.9	6.3
Banda Sea	2007-12-15	08:03:16.44	-7.61920	127.51460	181.0	6.0
S_Sandwich	2008-01-12	08:32:46.29	-56.4847	-27.31110	79.20	5.2
Mexico	2008-02-12	12:50:19.71	16.43150	-94.24270	85.70	6.5
S_Sandwich	2008-04-14	09:45:16.97	-56.0769	-28.09660	112.3	6.0
Fiji_Is	2008-04-18	20:39:07.79	-17.3916	-179.0091	556.2	6.3
BandaSea	2008-06-06	13:42:49.06	-7.55920	127.89660	121.7	6.0
Fiji_Is	2008-06-15	01:13:10.47	-17.7364	-179.7135	588.6	5.9
S_Sandwich	2008-07-01	01:54:41.00	-58.1210	-21.88200	10.00	5.6
S_Sandwich	2008-07-30	20:15:12.61	-59.7161	-27.88180	136.2	5.5
HinduKush	2008-09-06	05:47:40.00	36.44830	70.917200	192.0	5.8
S_Bolivia	2008-10-12	20:55:41.91	-20.1808	-64.98210	357.8	6.2
S_Sandwich	2008-10-19	02:56:07.77	-56.0078	-28.17420	101.0	5.1
Minahassa	2008-10-20	04:54:20.37	0.140100	120.69880	102.9	5.9
Tonga_Is	2008-10-22	12:55:57.82	-18.4657	-175.4010	234.8	6.4
Minahassa	2008-12-11	21:40:53.01	0.098400	123.56720	141.0	5.9
Fiji_Is	2008-12-17	10:55:59.93	-17.8607	-178.3674	531.6	5.8
Mindanao	2008-12-25	03:20:29.56	5.776000	125.52330	209.3	6.3
Afghanistan	2008-12-29	03:37:42.13	36.41200	71.089000	158.6	5.8
HinduKush	2009-01-04	23:12:59.97	36.50180	70.910500	194.6	5.7
BandaSea	2009-01-22	20:16:34.58	-7.31920	128.55870	149.5	6.1
Alaska	2009-01-24	18:09:49.94	59.55160	-153.1218	100.0	5.7
Fiji_Is	2009-01-26	11:54:40.70	-17.8406	-178.5160	595.0	5.8
W_Brazil	2009-01-29	22:28:06.02	-8.13680	-71.48380	610.0	5.6
Chile_Arg	2009-02-16	02:22:48.59	-29.0951	-69.83410	81.60	5.4
Fiji_Is	2009-02-20	01:46:18.36	-18.4361	-178.6623	522.2	5.6
Argentina	2009-04-03	17:54:47.98	-27.9029	-66.58710	155.5	5.7
S_Peru	2009-07-12	06:12:47.35	-15.0411	-70.53540	198.7	6.1
Fiji_Is	2009-08-15	23:30:19.22	-19.8755	-178.1389	609.4	5.6
Kermadec	2009-09-02	18:00:10.24	-29.2231	-178.8103	262.4	6.1
Kyushu	2009-09-03	13:26:18.24	31.17970	130.18260	167.2	6.2
Myanmar	2009-09-03	19:51:07.63	24.35550	94.702300	104.3	5.9
S_Peru	2009-09-05	03:58:39.10	-15.2524	-70.38390	208.6	5.8
Peru_Bol	2009-09-30	19:03:17.14	-15.6943	-69.44620	257.3	5.9
Afghanistan	2009-10-22	19:51:27.60	36.51870	71.012100	188.6	6.1
HinduKush	2009-10-29	17:44:32.11	36.38220	70.76840	213.9	6.2
Santa_Cruz	2009-10-31	19:09:51.60	-11.4345	166.47140	137.5	5.9
Kermadec	2009-11-22	22:47:27.73	-31.5501	179.55940	434.9	6.2
OhotskSea	2009-12-10	02:30:52.14	53.39870	152.79850	653.2	6.3
Andreanof	2009-12-17	20:01:21.90	51.43850	-179.9962	39.40	5.8
Primorye	2009-12-24	00:23:33.94	42.23750	134.77210	395.7	6.3
S_Sandwich	2010-02-03	02:18:28.23	-59.0240	-25.70800	51.50	5.2
S_Fiji	2010-02-07	22:28:20.61	-23.3150	-179.8153	545.5	5.9
BandaSea	2010-02-15	21:51:48.17	-7.26790	128.76660	134.7	6.2
Vanuatu	2010-03-04	14:02:28.85	-13.6248	167.23260	189.6	6.5
N_Chile	2010-03-04	22:39:25.71	-22.2651	-68.45790	108.4	6.3
Mariana	2010-03-08	09:47:10.58	19.35140	144.75990	446.9	6.1
S_Sandwich	2010-03-11	06:22:18.66	-57.2815	-27.93250	307.5	5.6

BandaSea	2010-03-26	10:39:02.80	-6.24330	130.30200	127.5	5.7
Argentina	2010-04-09	22:23:03.06	-28.5860	-68.19950	117.5	5.8
Fiji_Is	2010-04-10	16:54:24.07	-20.1887	-176.1483	272.7	5.9
Spain_	2010-04-11	22:08:11.32	37.00750	-3.476400	619.6	6.3
C_Peru	2010-05-23	22:46:51.68	-14.0000	-74.43190	102.6	6.1
W_Brazil	2010-05-24	16:18:28.81	-8.11520	-71.64120	582.1	6.5
Andaman	2010-05-31	19:51:47.69	11.21090	93.651900	131.6	6.5
S_Sandwich	2010-06-02	01:49:04.78	-57.4416	-26.49100	135.8	5.9
Fiji_Is	2010-06-22	22:16:21.09	-19.2325	-177.4436	571.7	5.8
N.Chile	2010-07-12	00:11:20.06	-22.2789	-68.31590	109.4	6.2
Vanuatu	2010-08-10	23:18:32.30	-14.4649	167.28710	196.1	5.9
PapuaNG	2010-08-15	15:09:29.57	-5.78250	148.37520	180.8	6.3
Fiji_Is	2010-08-16	19:35:48.46	-20.8416	-178.7564	600.8	6.2
C_Peru	2010-09-13	07:15:49.23	-14.6530	-70.84290	176.2	5.8
HinduKush	2010-09-17	19:21:15.20	36.53520	70.969500	215.4	6.2
S_Sandwich	2010-10-05	23:42:33.62	-57.2865	-24.41760	10.0	5.1
Solomon	2010-12-13	01:14:42.83	-6.52650	155.69590	144.8	6.2
S_Fiji	2010-12-28	08:34:17.10	-23.4129	-179.7154	550.9	6.3
Loyalty	2011-01-05	06:46:15.38	-22.2946	171.68350	123.2	6.1
Tonga_Is	2011-02-11	17:57:56.17	-20.8515	-175.5845	87.00	6.1
N_Chile	2011-03-06	12:31:58.50	-18.2064	-69.50060	114.5	6.3
S_Sandwich	2011-03-06	14:32:36.94	-56.3864	-27.02530	92.00	6.5
BaliSea	2011-03-10	17:08:37.32	-6.85670	116.73000	518.6	6.6
NETaiwan	2011-04-16	01:11:10.88	25.36090	124.03100	139.7	5.8
StdEstero	2011-04-17	01:58:49.47	-27.5503	-63.30640	562.1	5.8
HinduKush	2011-05-14	21:07:21.81	36.42570	70.723100	207.6	6.0
S_Sandwich	2011-05-21	00:16:27.12	-56.1533	-27.18220	64.20	5.9
Peru_Bol	2011-06-08	03:06:21.81	-17.2111	-69.68810	141.4	5.9
Minahassa	2011-07-08	05:22:40.56	0.108100	123.31960	160.4	5.4
Fiji_Is	2011-07-22	06:56:39.63	-20.2978	-178.4441	602.9	5.9
S_Fiji	2011-07-29	07:42:22.56	-23.7284	179.82520	522.8	6.7
S_Fiji	2011-08-02	19:18:47.32	-23.9510	179.13100	535.4	5.7
Ecuador	2011-08-15	02:53:17.82	-1.89990	-76.99880	174.2	5.7
Fiji_Is	2011-08-19	03:54:26.50	-16.5461	-176.8522	405.9	6.2
Vanuatu	2011-08-25	10:39:53.63	-13.6631	167.03050	118.4	5.9
StdEstero	2011-09-02	13:47:10.70	-28.4135	-63.13600	592.7	6.7
Hokkaido	2011-10-21	08:02:37.62	43.87290	142.53150	189.0	6.2
Afghanistan	2011-11-07	11:59:31.77	36.50880	71.088700	218.5	5.7
Nicaragua	2011-11-07	22:35:25.32	11.69180	-85.78380	177.2	6.0
Minahassa	2011-11-15	10:43:51.00	-0.10920	121.91650	271.6	5.8
Myanmar	2011-11-21	03:15:42.53	24.93060	95.166400	118.9	5.8
BaliSea	2011-11-28	09:13:11.41	-7.03370	116.96480	623.6	5.8
S_Sandwich	2011-12-11	09:54:56.48	-56.0490	-28.21350	130.6	6.3
Minahassa	2011-12-13	07:52:12.03	0.047300	123.06310	167.4	6.1
StdEstero	2012-03-05	07:46:09.33	-28.2579	-63.29160	551.9	6.1
Tonga_Is	2012-04-28	10:08:07.22	-18.7293	-174.6864	132.5	6.7
Peru_Brazil	2012-05-14	10:00:39.80	-17.7968	-69.79260	107.7	6.3
PapuaNG	2012-05-16	00:59:33.33	-5.60700	149.68850	149.7	5.8
StdEstero	2012-05-28	05:07:23.52	-28.0210	-63.11340	591.1	6.7
Salta_Arg	2012-06-02	07:52:54.60	-22.1017	-63.65740	539.4	6.0
Fiji_Is	2012-06-07	09:01:50.29	-20.1786	-176.2195	262.7	5.9
N_Sumatera	2012-06-23	04:34:53.60	3.002200	97.911600	101.9	6.1
Fiji_Is	2012-07-18	08:09:46.16	-20.8493	-178.4092	538.9	5.9
N_Molucca	2012-08-26	15:05:37.16	2.198300	126.86960	90.90	6.6
Loyalty	2012-10-23	09:39:30.37	-22.2730	171.68920	122.8	5.9
C_Peru	2012-11-10	14:57:50.21	-8.93670	-75.08840	128.6	6.0
Salta_Arg	2012-11-22	13:07:11.88	-22.7006	-63.62650	535.3	5.9
Solomon	2013-02-08	17:59:53.69	-6.25030	154.84030	93.9	5.7
NewZeeland	2013-02-16	05:16:16.76	-36.2346	178.02950	198.4	5.8
S_Sandwich	2013-03-19	03:29:02.7	-58.9815	-24.56970	52.00	6.0
Guatemala	2013-03-25	23:02:12.52	14.59050	-90.47720	193.7	6.2
E_Russia	2013-04-05	13:00:02.36	42.73590	131.06400	571.3	6.3
Vanuatu	2013-04-13	22:49:49.20	-19.1030	169.65240	269.3	6.0
S_Peru	2013-05-14	23:39:16.52	-15.6814	-73.16380	111.0	5.4
Okhotsk	2013-05-24	14:56:31.60	52.13570	151.56880	632.0	6.7
S_Sandwich	2013-06-09	00:21:35.30	-58.7857	-25.36250	40.40	5.3

NewBritain	2013-08-30	02:11:02.89	-4.44950	151.62280	211.0	5.9
Philippine	2013-09-06	11:33:54.79	20.18620	122.33360	189.4	6.0
FloresSea	2013-09-21	01:39:15.48	-7.26790	119.95080	550.1	6.1
E_Kamchatka	2013-11-12	07:03:52.47	54.67690	162.14320	60.50	6.5
Mariana	2013-11-19	17:00:45.01	18.46290	145.16500	531.1	6.0
Fiji_Is	2013-11-23	07:48:32.02	-17.1613	-176.5188	370.1	6.5
Fiji_Is	2013-12-01	10:01:31.81	-17.5737	-178.2609	534.8	5.7
Vanuatu	2014-01-01	16:03:29.77	-13.8767	167.27230	194.8	6.5
S_Sandwich	2014-02-01	03:58:43.99	-56.8269	-27.33910	130.0	6.2
S_Fiji	2014-03-26	03:29:35.00	-26.1690	179.28800	495.0	6.4
Solomon	2014-03-27	03:49:42.00	-12.0990	166.58900	98.00	6.0
SE_Loyalty	2014-05-01	06:36:35.55	-21.4542	170.35460	106.0	6.7
S_Fiji	2014-05-04	09:15:52.88	-24.6108	179.08560	527.0	6.6
S_Fiji	2014-05-04	09:25:15.00	-25.8070	178.24000	634.2	6.3
N_Mariana	2014-05-23	21:20:06.00	18.94900	145.04500	558.1	5.7
DominicanR	2014-05-28	21:15:06.00	18.04500	-68.35100	90.00	5.8
Alaska	2014-06-23	22:59:51.00	51.95900	178.58400	106.6	6.0
RatIsls	2014-07-03	19:06:48.00	52.12500	178.46500	116.1	5.7
C_Peru	2014-08-24	23:21:45.52	-14.5980	-73.57140	101.0	6.8
S_Sandwich	2014-09-22	16:01:42.23	-56.0228	-27.77920	109.3	5.8
Jujuy_Arg	2014-09-24	11:16:15.00	-23.8010	-66.63200	224.0	6.2
S_Alaska	2014-09-25	17:51:17.00	61.94500	-151.8160	108.9	6.2
Taiwan	2014-12-10	21:03:39.00	25.54000	122.45000	256.0	6.1
Fiji_Is	2014-12-12	20:22:35.00	-18.9040	-176.4440	316.4	5.8
Fiji_Is	2014-12-30	21:17:23.00	-20.3260	-178.5640	599.3	6.0
Fiji_Is	2015-01-28	02:43:19.00	-20.9660	-178.3030	484.1	6.2
Mendoza	2015-02-02	10:49:48.00	-32.7180	-67.12300	172.0	6.3
N_Colombia	2015-03-10	20:55:44.00	6.776000	-72.98700	155.0	6.2
PapuaNG	2015-03-16	03:00:05.00	-4.07400	152.02900	196.0	5.9
N_Chile	2015-03-23	04:51:38.00	-18.3530	-69.16600	130.0	6.4
Fiji_Is	2015-04-02	04:10:10.00	-17.8610	-178.5830	560.0	5.9
Fiji_Is	2015-04-28	16:39:39.00	-20.8870	-178.6330	581.0	6.1
S_Sumatera	2015-05-15	20:26:56.00	-2.54200	102.21900	151.0	6.0
S_Sandwich	2015-05-24	21:06:41.14	-59.6530	-26.45460	33.00	5.8
N_Chile	2015-06-10	13:52:09.00	-22.4000	-68.43200	124.0	6.0
S_Sandwich	2015-06-20	05:32:08.00	-59.6290	-26.50300	50.10	5.7
S_Fiji	2015-06-20	23:39:09.51	-23.5295	-177.0845	151.0	5.9
Fiji_Is	2015-06-21	21:28:16.00	-20.4310	-178.3280	562.6	6.0
BoninSea	2015-06-23	12:18:30.00	27.73800	139.72500	460.0	6.5
Fiji_Is	2015-07-17	18:49:53.00	-18.1210	-178.1890	536.1	5.8
S_Alaska	2015-07-29	02:35:59.00	59.89400	-153.1960	119.3	6.3
Mexic_	2015-08-05	09:13:24.00	16.14100	-93.66400	100.8	5.7
S_Fiji	2015-08-06	23:59:46.00	-26.4580	-178.2510	269.0	6.0
S_Fiji	2015-09-07	08:46:09.00	-24.2430	179.12800	535.0	5.8
S_Sandwich	2015-09-30	16:06:52.00	-56.1900	-27.72000	101.0	5.7
N_Chile	2015-11-01	15:16:17.00	-23.2470	-68.41700	111.0	5.8
Mariana	2015-11-24	13:21:35.00	18.77900	145.26600	586.9	6.0
Peru_Brazil	2015-11-26	05:45:18.40	-9.18250	-71.25740	602.8	6.7
NE_China	2016-01-02	04:22:19.00	44.80700	129.94100	585.5	5.8
Tonga_Is	2016-01-13	05:56:02.00	-15.1700	-174.8750	263.0	5.9
S_Sandwich	2016-02-18	01:07:11.53	-56.2549	-27.56610	99.00	5.7
Fiji_Is	2016-02-20	15:51:23.00	-21.9560	-179.4070	582.6	5.8
S_Sumatera	2016-05-02	04:21:24.00	-5.02600	104.62700	117.0	5.7
Chile_Arg	2016-06-18	17:29:46.00	-24.1470	-67.15300	186.0	5.7
PapuaNG	2016-06-21	17:12:07.00	-3.42000	151.88000	354.0	6.3
NW_Kuril	2016-07-23	01:00:20.00	47.69800	146.92100	408.0	5.8
Argentina	2016-08-04	14:15:12.00	-22.3340	-66.00800	270.0	6.2
N_Peru	2016-09-10	10:08:20.00	-5.57300	-76.95400	121.0	6.1
PapuaNG	2016-10-15	08:03:38.00	-4.27400	150.36100	442.0	6.3
Tyrrhenian	2016-10-28	20:02:49.00	39.38800	13.521000	457.9	5.8
BismarkSea	2016-11-09	14:50:59.00	-4.74000	149.87100	448.8	5.9
Argentina	2016-11-13	14:01:01.00	-28.8650	-67.45700	109.1	5.7
Argentina	2016-11-20	20:57:44.00	-31.6226	-68.62590	108.0	6.4
Talaud	2016-12-04	05:24:05.00	4.505000	127.83400	139.0	5.7
BandaSea	2016-12-05	01:13:04.00	-7.31600	123.38000	526.0	6.3
Solomon	2016-12-10	16:24:24.00	-5.65900	154.47300	142.6	6.0

Peru_Brazil	2016-12-18	13:30:10.00	-9.97400	-70.97000	612.0	6.4
S_Fiji	2017-01-02	13:14:02.00	-23.2510	179.23800	551.6	6.3
S_Bolivia	2017-02-21	14:09:04.00	-19.2870	-63.92100	596.0	6.5
Vanuatu	2017-04-05	22:17:42.00	-18.9890	169.57700	248.3	5.6
Mexico	2017-05-20	17:58:46.00	17.14000	-93.97100	144.0	5.7
S.Peru	2017-06-24	16:09:11.00	-15.4710	-73.45200	69.00	5.8
Argentina	2017-08-01	06:13:54.00	-28.5550	-68.70600	85.00	5.6
Chile_Arg	2017-08-02	07:15:14.00	-33.2090	-70.63500	88.00	5.4
NW_Ryukyu	2017-08-16	12:51:25.00	28.64780	127.92380	196.5	5.7
Fiji_Is	2017-08-19	02:00:52.00	-17.9654	-178.8503	538.6	6.5
S_Sumatera	2017-08-31	17:06:57.00	-1.12300	99.708000	49.00	6.3
S_Sandwich	2017-09-04	08:07:35.00	-57.7890	-25.57800	35.00	6.1
BoninSea	2017-09-07	17:26:49.00	27.76430	139.81120	448.3	6.1
Volcano	2017-09-11	17:35:09.00	23.95290	142.33730	35.00	5.7
Mariana	2017-09-14	17:41:28.00	18.66990	145.73200	173.3	5.8
Vanuatu	2017-09-20	20:09:49.00	-18.7978	169.09470	200.2	6.4
JavaSea	2017-09-20	23:59:24.00	-6.14100	113.00280	588.6	5.7
N_Chile	2017-10-10	06:32:20.00	-18.5212	-69.64110	82.43	6.3
